# Development speed of sibling embryo positively reflects live birth rate after fresh day 3 embryo transfer

**DOI:** 10.1038/s41598-023-33573-6

**Published:** 2023-04-19

**Authors:** Xue Wang, Yaling Xiao, Yuanzheng Zhou, Hanbi Wang

**Affiliations:** Department of Gynaecology Endocrine and Reproductive Centre, State Key Laboratory of Complex Severe and Rare Disease, Peking Union Medical College Hospital, Chinese Academy of Medical Sciences and Peking Union Medical College, Beijing, 100730 China

**Keywords:** Reproductive disorders, Reproductive signs and symptoms

## Abstract

The ability of sibling embryos to form blastocysts may reflect the developmental potential of the embryos that were transferred into the uterus. The purpose of the study was to investigate whether the development speed of sibling embryos positively reflects the live birth rate following fresh embryo transfer. We examined 1262 cycles of women who underwent day 3 (D3) cleavage embryo transfer in the Peking Union Medical College Hospital in 2015–2020, who were divided into three groups (D5, D5 + D6, and D6) according to blastocyst formation. The live birth rate in patients with blastocysts that formed on D6 was significantly lower than the other two groups (36.1%, 45.6% and 44.7%, *P* < *0.05*). For women with blastocysts that formed on D6, the live birth rate was higher in those with more good quality blastocysts than poor-quality blastocysts (42.4 vs 32.3%, *P* < *0.05*). Multiple regression analysis showed that the blastocyst development speed of sibling embryos was an independent factor affecting live birth after fresh embryo transfer (*P* < *0.05*). We concluded that the blastocyst development speed of sibling embryos may reflect live birth rate following the transfer of D3 cleavage embryos.

## Introduction

In recent years, more infertile women seek in vitro fertilization (IVF) and embryo transfer to achieve live birth. The success of IVF can be affected by many factors, including patient age, ovary status, ovulation protocol, and embryo quality^1^. Embryo quality is particularly important for IVF success and therefore, critical. There has been recent technological developments in the methods of embryo evaluation, including time-lapse imaging^2,3^, embryo metabolomics^4^, and preimplantation genetic testing for aneuploidy^5^. However, classical morphological evaluation is still the gold standard for predicting embryos with high implantation potential and achieving high live birth probability^6,7^. With the improvement of blastocyst culture technology, a growing number of centres choose blastocyst culture or single blastocyst transfer^8^. Embryos with high developmental potential can be selected through blastocyst culture, in which the blastocyst formation rate reflects the developmental potential of the embryos^9^. However, not all cleavage-stage embryos can form blastocysts and thus, most centres still use cleavage-stage transfer and blastocyst culture for surplus or sibling embryos obtained after transfer during the assisted reproductive technology procedure.

Several studies suggest that the number of surplus embryos positively correlates with live birth rate^10,11^. Moreover, after the transfer of day 3 (D3) cleavage embryos, the blastocyst formation rate of sibling embryos from pregnant patients is significantly higher than that of embryos from non-pregnant patients^12^. These findings suggest that if one embryo has a high developmental potential, other embryos from the same oocyte retrieval cycle will also have high developmental potential. Thus, the ability of sibling embryos to form blastocysts may be a prognostic factor for clinical outcomes following D3 cleavage embryo transfer^12^. Indeed, many studies have suggested that the implantation and clinical pregnancy rates in groups in which sibling embryos formed blastocysts were reported to be significantly higher than in those in which sibling embryos did not form blastocysts; consequently, associating the ability to form blastocysts with higher live birth rates^13–23^.

Although the time of blastocyst formation varies, most blastocysts are formed on day 5 (D5), day (D6), or day 7 (D7) after fertilisation^1,13,14^. Some studies found that the survival ability of embryos with a faster development speed was high; whereas, slow development was associated with a lower pregnancy rate and suggested that embryonic development speed may be predictive of the likelihood of successful implantation after transferred blastocysts^1,14^. We hypothesised that the blastulation day of sibling embryos could potentially serve as a probability marker for live birth after fresh embryo transfer. However, to the best of our knowledge, the relationship between blastulation day of sibling embryos and clinical outcomes of D3 cleavage embryo transfer has not been examined. Therefore, we aimed to determine whether the blastocyst development speed of sibling embryos positively corelated with the live birth rate after the transfer of fresh D3 cleavage embryos.

## Results

In this study, we retrospectively analysed the data from 1262 women with a mean age of 33.2 ± 3.2 years. The implantation, clinical pregnancy, and live birth rates were 34.9%, 53.7%, and 42.2%, respectively. The average number of blastocysts from sibling embryos was 3.0 ± 2.0, while the average ages of patients in the groups D5, D5 + D6, and D6 were 33.3 ± 3.1, 33.1 ± 3.3, and 33.6 ± 3.2 years, respectively (*P* = 0.06). The demographic characteristics of the patients included in the study are shown in Table 1. There were no significant differences in the baseline parameters, such as basal follicle-stimulating hormone and oestradiol levels, body mass index, endometrial thickness, and the types of infertility among the groups (*P* > 0.05). The protocol for ovarian stimulation and the insemination method were also similar among the three groups (*P* > 0.05). The number of oocytes retrieved and 2-pronuclear stage (2PN) zygotes were significantly different among the groups D5, D5 + D6, and D6 (8.9 ± 3.1, 11.3 ± 3.1, and 9.0 ± 3.0; *P* < 0.01 and 7.2 ± 2.8, 9.7 ± 3.0, and 7.3 ± 2.9; *P* < 0.01), respectively. The rate of good embryos transferred on D3 was significantly lower in group D6 than in groups D5 + D6 and D5 (37.9%, 64.75%, and 52.6%, respectively, P < 0.01).

**Table 1:** Demographic characteristics of patients with different blastocyst development speeds.

Parameter	Group D5	Group D5 + D6	Group D6	*P*-value
No. of cycles	322	522	418	
Age (years)	33.3 ± 3.1	33.1 ± 3.3	33.6 ± 3.2	0.06
Duration of sterility (years)	3.8 ± 2.4	3.8 ± 2.5	3.9 ± 2.5	0.89
Primary infertility % (n)	56.5 (182/322)	58.4 (305/522)	62.4 (261/418)	0.24
Basal FSH (IU/L)	7.7 ± 2.8	7.6 ± 2.8	7.6 ± 2.2	0.78
Basal E2 (pg/ml)	47.8 ± 20.0	48.1 ± 27.3	48.5 ± 31.0	0.94
BMI	21.9 ± 2.9	22.0 ± 2.9	21.9 ± 3.0	0.64
Endometrial thickness (cm)	11.4 ± 1.7	11.5 ± 1.8	11.7 ± 1.8	0.12
Cause of infertility				
Oviduct factor	30.1 (97/322)	36.0 (188/522)	34.2 (143/418)	0.21
Endometriosis	22.7 (73/322)	24.3 (127/522)	19.4 (81/418)	0.19
Male factor	37.6 (121/322)	43.1 (225/522)	41.6 (174/418)	0.28
Abnormal ovulation	22.7 (73/322)	20.7 (108/522)	26.1 (109/418)	0.15
GnRH antagonist (%)	36.6 (118/322)	32.6 (170/522)	30.9 (129/418)	0.24
ICSI (%)	28.3 (91/322)	25.7 (134/522)	27.0 (113/418)	0.70
Number of oocytes retrieved	8.9 ± 3.1	11.3 ± 3.1	9.0 ± 3.0	< 0.01
Number of 2PN zygotes	7.2 ± 2.8	9.7 ± 3.0	7.3 ± 2.9	< 0.01
Rate of good embryos transferred (%) on D3	52.6 (339/644)	64.7 (675/1044)	37.9 (317/836)	< 0.01

*BMI* body mass index, *D5%* number of blastocysts formed on D5 × 100%/total number of blastocysts, *FSH* follicle-stimulating hormone, *GnRH* gonadotropin-releasing hormone, *ICSI* intracytoplasmic sperm injections, *IVF* in vitro fertilisation, *PN* pronucleus.

Differences were considered statistically significant if *P* < 0.05.

There were no significant differences in the implantation rates and clinical pregnancy rates among the three groups (Table 2; P > 0.05). However, the live birth rate in group D6 (36.1%) was significantly lower than that in groups D5 + D6 and D5 (45.6% and 44.7%, respectively; *P* < 0.01). The miscarriage rate in group D6 was significantly higher than that in the other two groups (P = 0.047).

**Table 2:** Clinical outcomes after D3 cleavage embryo transfer in patients with different blastocyst development speeds.

Parameter	Group D5	Group D5 + D6	Group D6	*P*-value
No. of cycles	322	522	418	
Implantation rate (%)	36.0 (232/644)	36.4 (380/1044)	32.1 (268/836)	0.11
Clinical pregnancy rate (%)	56.2 (181/322)	55.6 (290/522)	49.5 (207/418)	0.11
Live birth rate (%)	44.7 (144/322)	45.6 (238/522)	36.1 (151/418)	0.008
Miscarriage rate (%)	20.4 (37/181)	17.9 (52/290)	27.1 (56/207)	0.047

Differences were considered statistically significant if *P* < 0.05.

### Subgroup analysis

The rate of high-quality blastocysts in group D5 was significantly higher than that in group D6 (67.7% vs. 37.8%, *P* < 0.01). For women with blastocysts that formed at a higher development speed (D5), the implantation, clinical pregnancy, and live birth rates were similar between subgroups D5-1 and D5-2 (*P* > 0.05). However, for women with blastocysts that formed at a lower development speed (D6), the implantation rates, clinical pregnancy rate and live birth in subgroup D6-1 (36.1%, 56.3% and 42.4%) were significantly higher than those in subgroup D6–2 (29.6%, 45.4% and 32.3%, respectively; *P* < 0.05; Table 3).

**Table 3:** Clinical outcomes after D3 cleavage embryo transfer in patients with different blastocyst quality.

Variable	Group D5			Group D6		
	D5-1	D5-2	*P*-value	D6-1	D6-2	*P*-value
No. of cycles	218	104		158	260	
Implantation rate (%)	30.8 (159/516)	35.1 (73/208)	0.26	36.1 (114/316)	29.6 (154/520)	0.05
Clinical pregnancy rate (%)	55.5 (121/218)	58.7 (61/104)	0.59	56.3 (89/158)	45.4 (118/260)	0.03
Live birth rate (%)	44.0 (96/218)	46.2 (48/104)	0.72	42.4 (67/158)	32.3 (84/260)	0.04
Miscarriage rate (%)	20.7 (25/121)	21.3 (13/61)	0.92	24.7 (22/89)	28.8 (34/118)	0.51

Differences were considered statistically significant if *P* < 0.05.

### Logistic regression analysis of factors affecting live birth rate

Multiple logistic regression analysis showed that endometriosis and the development speed of sibling embryos were independent factors that affected the live birth rate after the transfer of D3 cleavage embryos (Table 4). Compared with those in the reference group (group D6), the live birth rate was significantly higher in group D5 (odds ratio [OR] = 1.409 and 95% CI 1.013–1.958; P = 0.04). Moreover, the live birth rate of patients whose blastocysts formed on both D5 and D6 was 1.650-fold higher than that of patients whose blastocysts formed only on D6 (*P* = 0.006).

**Table 4:** Logistic regression analysis of factors that may affect live birth rate.

Variable	LR		
	OR	95% CI	*P*-value
Age	0.613	0.970–1.052	0.613
Basal FSH	1.000	0.950–1.050	0.962
ICSI	1.065	0.770–1.473	0.702
GnRH antagonist	0.802	0.616–1.046	0.103
Duration of sterility	1.032	0.980–1.086	0.231
Abnormal ovulation	1.075	0.766–1.508	0.677
Endometriosis	1.555	1.105–2.187	0.011
No. of oocytes	0.940	0.872–1.015	0.113
No. of 2PN	1.014	0.931–1.104	0.756
No. of blastocyst	1.012	0.919–1.114	0.81
No. of good embryos transferred on D3	0.941	0.806–1.098	0.439
Blastulation day			0.016
D6 blastocyst	Reference		
D6 + D5 blastocyst	1.650	1.154–2.358	0.006
D5 blastocyst	1.409	1.013–1.958	0.041

*CI* confidence interval, *ICSI* intracytoplasmic sperm injections, *FSH* follicle-stimulating hormone, *OR* odds ratio, *PN* pronucleus.

Differences were considered statistically significant if *P* < 0.05.

## Discussion

We found that when more embryos were produced in one oocyte retrieval cycle, the ability of sibling embryos to form blastocysts predicted the pregnancy outcome of the current cycle. Mackenna et al. showed that the clinical pregnancy rate (55.8% vs*.* 40.6%; *P* < 0.01), live birth rate (50.0% vs*.* 37.2%; *P* < 0.05), and implantation success rate (34.2% vs*.* 23.7%; *P* < 0.01) in patients with blastocyst formation were significantly higher than those in patients without blastocyst formation^10^. Hence, the authors suggested that blastocyst formation of the remaining embryos not only predicts the outcome of D3 embryo transfer, but also provides valuable reference information for preserved embryo transfer in the future. Other studies found that the number of blastocysts formed by the remaining embryos significantly and positively correlated with implantation and live birth rates^18,20^. This showed that blastocyst formation was indeed a good predictor for clinical outcome^24^. The day of blastocyst formation can vary from D5 to D7, so we asked whether the blastocyst development speed of sibling embryos could predict the clinical outcome of a fresh embryo transfer.

Previous studies report that the clinical outcomes of a D5 blastocyst transfer are better than those of a D6 blastocyst transfer, irrespective of the use of fresh or frozen cycles^1,14^. Therefore, we hypothesised that the speed of blastocyst formation from sibling embryos correlates with live birth probability after D3 cleavage embryo transfer. We found significant differences in the live birth rate between the D5, D5 + D6, and D6 groups, as well as in the miscarriage rates after D3 cleavage embryo transfer. These results may be related to the lower rate of good quality embryos transferred at D3 in group D6. In addition, the age of the patients in group D6 was slightly older, although there was no significant difference among the groups. However, the multiple regression analysis showed that age, number of oocytes and the quality of embryos transferred at D3 were no longer risk factors for live birth rate. After adjusted for confounding factors, the development speed of sibling embryos still positively correlated with the live birth rate after fresh D3 cleavage embryo transfer. The live birth rates in groups D5 and D5 + D6 were similar, and significantly higher than those of group D6. These findings were consistent with the results from the blastocyst transfer cycles, which revealed that patients who received D5 blastocyst transfer had better clinical outcomes than those with D6 blastocysts. These difference between the two groups persisted even when the blastocysts were of similar quality^25^. Some studies suggest that the higher rate of aneuploidy and genetic abnormalities in retarded blastocysts result in clinical outcome differences between D5 and D6 groups^26,27^. Exploring the relationship between morphological and morphodynamic parameters, and euploid status of 1,730 blastocysts, Minasi et al. found that euploid embryos form expanded blastocysts more rapidly, with improved trophectoderm and inner cell mass, and higher proportions of normal chromosomes in the D5 blastocyst group, after patients were grouped by age. Moreover, D5 blastocyst transfer was hypothesised to increase the chances of selecting euploid blastocysts in women who did not undergo preimplantation genetic testing^27^. Therefore, our results suggested that the speed of blastocyst formation in sibling embryos could predict pregnancy outcome after D3 transfer.

Our results also showed that when the blastocyst development speed was faster (D5), the live birth rate after D3 cleavage transfer were similar between the two groups irrespective of the quality of blastocysts from the sibling embryos (D5-1vs. D5-2, *P* > 0.05). However, when sibling embryos developed slowly (D6), patients with a high proportion of good blastocysts (D6-1) had a significantly higher live birth rate after D3 transfer than those with a low proportion of good blastocysts (D6-2; 42.4% vs 32.3%, P < 0.05). This may be because euploid embryos are more likely to develop faster than aneuploid embryos, regardless of their quality. It has been reported that even in transferred euploid embryos, the clinical outcomes in women who received D6 blastocysts were worse than those in women who received D5 blastocysts^28^. While the difference in euploidy rate cannot explain why the speed of blastocyst development affects the clinical outcomes of embryos, it does suggest that other factors are likely involved, such as embryo metabolism or epigenetics. Blastocyst formation of sibling embryos likely represents the developmental capacity of the transferred embryos; the embryos that develop faster are more likely to form euploid blastocysts, suggesting that the embryos transferred on D3 could develop into euploid embryos in vivo and proceed to live birth. Overall, our study suggests that the more blastocysts formed on D5 from sibling embryos, the higher the live birth rate after D3 transfer, regardless of the blastocyst quality. However, in slower developing sibling embryos, the blastocyst quality predicted the pregnancy outcome after D3 transfer.

Our study highlights the importance of the development speed of sibling embryos on the clinical outcome of D3 fresh cleavage embryo transfer. These parameters may help retrospectively evaluate the clinical outcome of current transplantation cycle and predict the cumulative pregnancy rates in the future. The results of our study can also help clinicians make more accurate medical decisions in subsequent cycles. In addition, the formation of blastocysts from sibling embryos can help us better understand the causes of failed transfer cycles. For example, when sibling embryos display a high blastocyst development speed and high quality but unsuccessful D3 cleavage embryo transfer, factors other than embryo quality such as endometrial characteristics, luteal function support, endocrine and immune status, or embryologist skill must be considered when analysing the causes of implantation failure.

Our study had some limitations. First, the transferred D3 fresh embryos were selected using morphological criteria, which was a subjective procedure conducted by embryologists; therefore, the embryos with highest developmental potential may not have been consistently identified. These embryos would not be selected for transfer or cultured to blastocyst-stage. If D3 cleavage embryo transfer is unsuccessful even though sibling embryos displayed a high blastocyst development speed and high quality, we still cannot rule out the effect of embryo quality on the outcome. Second, this was a retrospective study, which could have been affected by biases and confounding factors. To diminish the impact of these factors on our analysis, we only included patients without uterine or endometrial disease and those who received the same number of transferred embryos. In addition, we minimised the impact of confounding factors by performing multiple regression analysis, which further validated our conclusions.

## Conclusions

Our study suggests that blastocyst development speed of sibling embryos positively correlated with live birth after fresh D3 embryo transfer. The blastocyst quality could also predict live birth when the sibling embryos formed blastocysts on D6. These findings provide a new alternative method for predicting the outcome of D3 embryo transfer, with exception to the morphological evaluation of embryo cleavage stage. The analysis of sibling embryo properties will help establish possible causes of embryo transfer failure and select optimal treatments, thereby increasing the chances of successful pregnancy in women undergoing further treatment cycles.

## Methods

### Study design and participants

**Figure 1:**
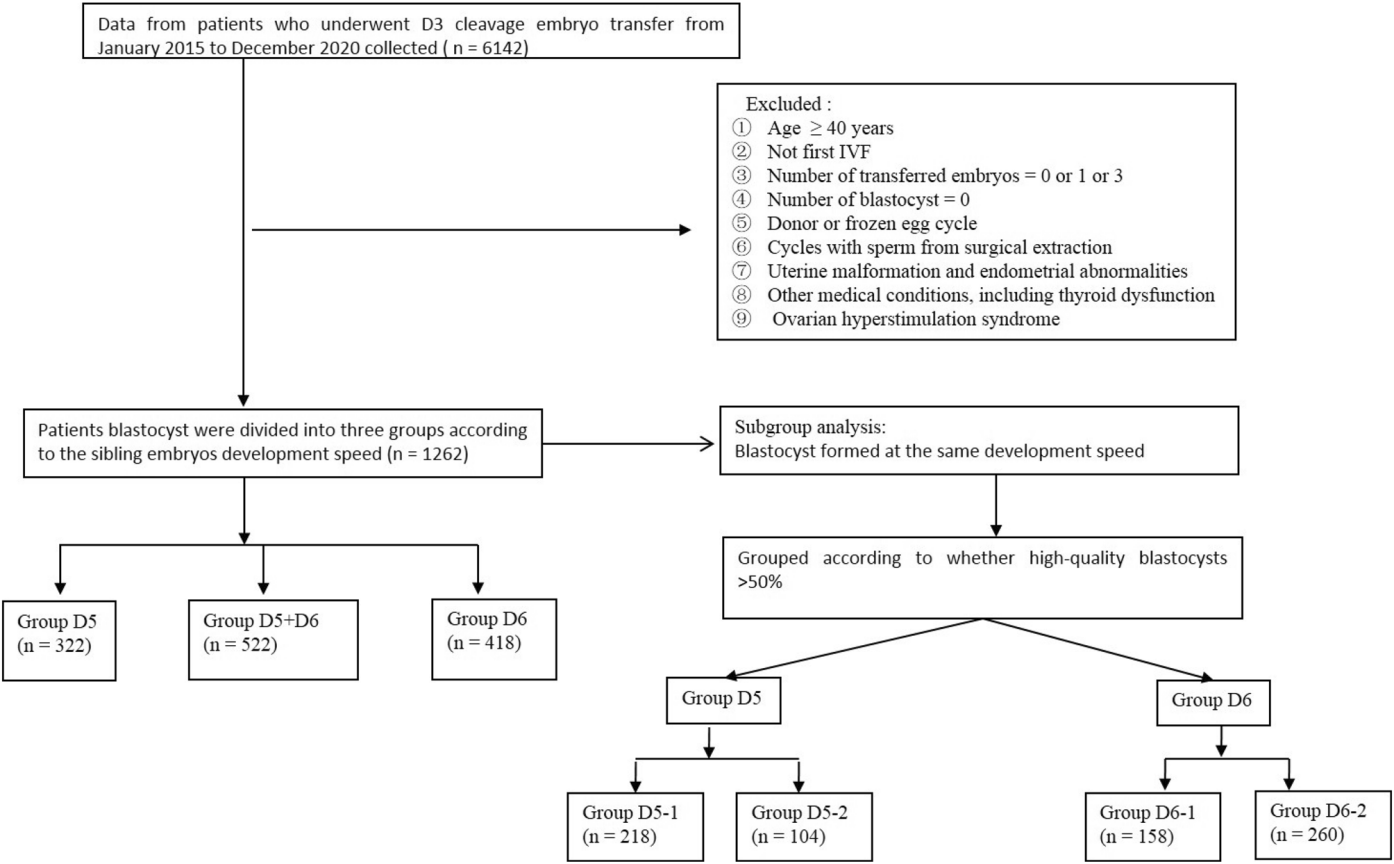
Flow chart of the patient inclusion process used in this study.

Records of women who underwent D3 cleavage embryo transfer between January 2015 and December 2020 in the Reproductive Centre of the Peking Union Medical College Hospital were retrospectively analysed. Women who met the following criteria were included: (1) age < 40 years, (2) first IVF, (3) number of transferred embryos = 2, and (4) number of blastocysts ≥ 1. The exclusion criteria were as follows: (1) donor or frozen egg cycle, (2) cycles with sperm from surgical extraction, (3) uterine malformation and endometrial abnormalities, (4) medical conditions including thyroid dysfunction, and (5) ovarian hyperstimulation syndrome (Fig. 1). This study was approved by the Ethics Committee of the Peking Union Medical College Hospital. All methods were performed in accordance with the relevant guidelines and regulations.

### Stimulation protocols and embryo culture

All patients underwent controlled ovarian stimulation using either a GnRH agonist or a flexible GnRH antagonist protocol. Briefly, ovarian stimulation was induced with recombinant follicle-stimulating hormone (Gonal F; Merck KGaA, Darmstadt, Germany). Next, 250 μg human chorionic gonadotropin (Azer; Merck KGaA) was administered to trigger final ovulation once two or more dominant follicles reached ≥ 18 mm in diameter. Oocyte retrieval was conducted transvaginally after 38 h. According to the quality of the available semen, either IVF or intracytoplasmic sperm injection was used for insemination. The 2PN embryos were observed 18–20 h after insemination and cultured in cleavage medium (G1, Vitrolife, Sweden) in an incubator (HERAcell 150i, Thermo, Germany) with 6% CO_2_, 5% O_2_, and 89% N_2_. Cleavage embryos were evaluated by cell number, fragmentation, and uniformity grade on D3 after fertilisation^29^. Embryos with 7–9 blastomeres that were symmetrical and evenly arranged with less than 10% fragmentation were classified as good-quality embryos. The two highest quality embryos were transferred into the uterus, and all remaining embryos were transferred into blastocyst culture medium (G2, Vitrolife) and continuously cultured as a group of 4–5 embryos in a drop until D5 or D6 in the in incubator with 6% CO_2_, 5% O_2_, and 89% N_2_. Blastocysts were evaluated using the Gardner score^30^, based on the degree of expansion of the blastocyst cavity, quality of the inner cell mass, and trophoblast cells. The degree of expansion of the blastocyst cavity was divided into stages 1–6. At stage 4, blastocysts showed full cavity expansion and zona pellucida thinning; at stage 5, blastocysts had partial trophoblasts growing from the zona pellucida; and at stage 6, blastocysts were completely detached from the zona pellucida. According to the previous experiences at our centre, we found the recovery rates of early blastocysts were not ideal, so we selected blastocysts at stage 4 or above for cryopreservation. The vitrification criteria for blastocysts require that the score of ICM is A or B and the TE is A or B or C. Blastocysts with an inner cell and trophoblast mass of A or B (grades: AA, AB, BA, and BB) were defined as high-quality embryos, whereas those with the BC grade were defined as embryos of poor quality. The rate of high-quality blastocyst was defined as the total number of high-quality blastocysts/total number of blastocysts) × 100%.

### Definition of groups

The patients were divided into three groups according to the day expanded blastocysts formed from the sibling embryos: group D5 had expanded blastocysts that formed on D5, group D5 + D6 had expanded blastocysts that formed on both D5 and D6, and group D6 had expanded blastocysts that formed on D6. To investigate whether the quality of blastocysts from sibling embryos affected the clinical outcomes, the patients whose blastocysts formed on the same day were grouped according to whether the high-quality blastocyst rate of formation was ≥ 50%. Therefore, groups D5 and D6 were further divided into two subgroups: groups D5-1 and D6-1, in which the rate of high-quality blastocyst formation was ≥ 50%, and groups D5-2 and D6-2, in which the rate of high-quality blastocyst formation was < 50%).

### Clinical outcome measurements

The primary goal was to explore the impact of the development speed of sibling embryos on the live birth rate after the transfer of D3 cleavage embryos. The impact of the blastulation day on clinical pregnancy and implantation rates was also determined. Biochemical pregnancies were identified by blood sampling two weeks after oocyte retrieval. Clinical pregnancies were confirmed by a vaginal ultrasound examination that showed one or more gestational sac with heartbeat four weeks after oocyte retrieval. Live birth was defined as delivery of a live baby after 28 weeks of gestation following embryo transfer.

### Statistical analysis

The SPSS 22.0 software (IBM Corp. Armonk, NY, USA) was used for all statistical analyses. The Kolmogorov–Smirnov test was performed to explore data distribution; if the data were normally distributed, the groups were compared by analysis of variance (ANOVA), otherwise, they were analysed by the Kruskal–Wallis *H*-test. The rates among groups were compared by the χ^2^ test. The multivariate logistic regression analysis was conducted to assess whether blastulation day was corelated to the live birth rate after adjusting for confounding factors. Effects were defined as statistically significant if* P* < 0.05.

### Ethics approval and consent to participate

This study was approved by the Ethics Committee of the Peking Union Medical College Hospital, Beijing, China (Approval No. zs-1214). The Ethics Committee waived the requirement for informed consent due to the retrospective nature of the study.

## Data Availability

All data generated or analysed during this study are included in the published article.
